# Green Synthesis of Nanomaterials

**DOI:** 10.3390/nano11082130

**Published:** 2021-08-21

**Authors:** Matthew Huston, Melissa DeBella, Maria DiBella, Anisha Gupta

**Affiliations:** 1 Internal Medicine-Infectious Disease, University of Michigan, Ann Arbor, MI 48109, USA; hustonma@umich.edu; 2 Department of Pharmaceutical Sciences, University of Saint Joseph, Hartford, CT 06117, USA; mdebella@usj.edu (M.D.); mdibella@usj.edu (M.D.)

**Keywords:** green synthesis, nanomaterials, plant, yeast, fungi

## Abstract

Nanotechnology is considered one of the paramount forefronts in science over the last decade. Its versatile implementations and fast-growing demand have paved the way for innovative measures for the synthesis of higher quality nanomaterials. In the early stages, traditional synthesis methods were utilized, and they relied on both carcinogenic chemicals and high energy input for production of nano-sized material. The pollution produced as a result of traditional synthesis methods induces a need for environmentally safer synthesis methods. As the downfalls of climate change become more abundant, the scientific community is persistently seeking solutions to combat the devastation caused by toxic production methods. Green methods for nanomaterial synthesis apply natural biological systems to nanomaterial production. The present review highlights the history of nanoparticle synthesis, starting with traditional methods and progressing towards green methods. Green synthesis is a method just as effective, if not more so, than traditional synthesis; it provides a sustainable approach to nanomaterial manufacturing by using naturally sourced starting materials and relying on low energy processes. The recent use of active molecules in natural biological systems such as bacteria, yeast, algae and fungi report successful results in the synthesis of various nanoparticle systems. Thus, the integration of green synthesis in scientific research and mass production provides a potential solution to the limitations of traditional synthesis methods.

## 1. Introduction

**Figure 1:**
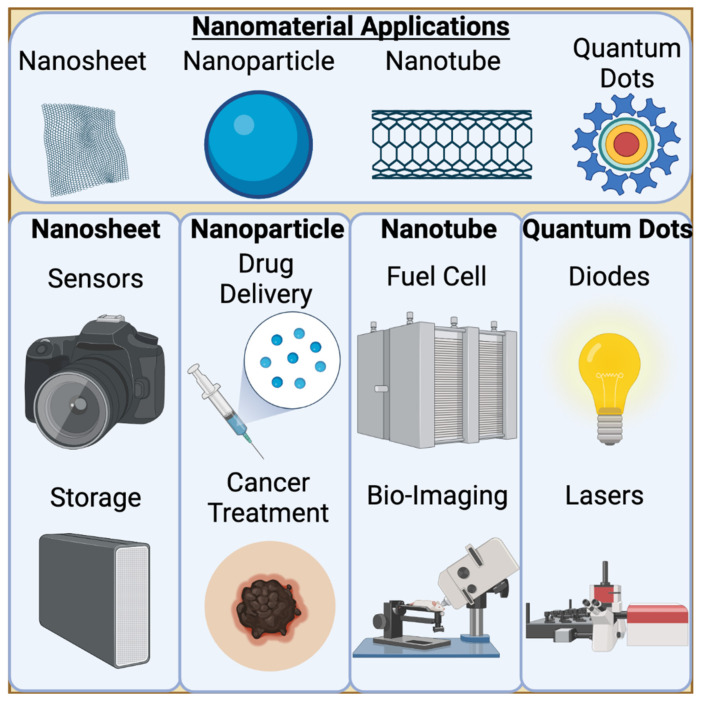
Applications of Nanomaterials. 0D and 1D nanomaterials have extensive applications throughout multiple sectors. Nanosheets, a 1D nanomaterial, are highly utilized in electronics as they can be highly conductive and sensitive. Nanoparticles are a 0D nanomaterial that are highly employed for drug delivery. Most notably, nanoparticle technology is used in the delivery of SARS-CoV-2 vaccines. Nanotubes are long, 1D nanomaterials that can vary in thickness depending on their application. For bio-imaging, single walled carbon nanotubes can be used to target specific tissues and emit a fluorescent signal bright enough to be detected. Finally, quantum dots, or buckyballs, vary slightly from nanoparticles in their composition. They are smaller and emit a light when excited, making them useful for lasers, amongst other applications.

Over the past few years, a large amount of attention has been directed towards nanotechnology. The classification of nano-sized technology encompasses materials between 1 and 100 nano-meters [1]. Though the size of the compound is what classifies it as a nanomaterial, its morphology and geometry also play a significant role in its characteristics. Nano-sized materials have applications in almost every sector including, but not limited to: electronics, agriculture, and medicine (Figure 1). Nanotechnology allows nanoparticles to revolutionize the materials designed for use, resulting in noteworthy improvements in thermal, mechanical, and barrier properties [2]. The fin^e-^combed development of various nanoparticle morphologies, such as spheres, rods, quantum dots and particles, allow for variety in applications and can arguably result in limitless opportunity for technological advancement [3].

**Figure 2:**
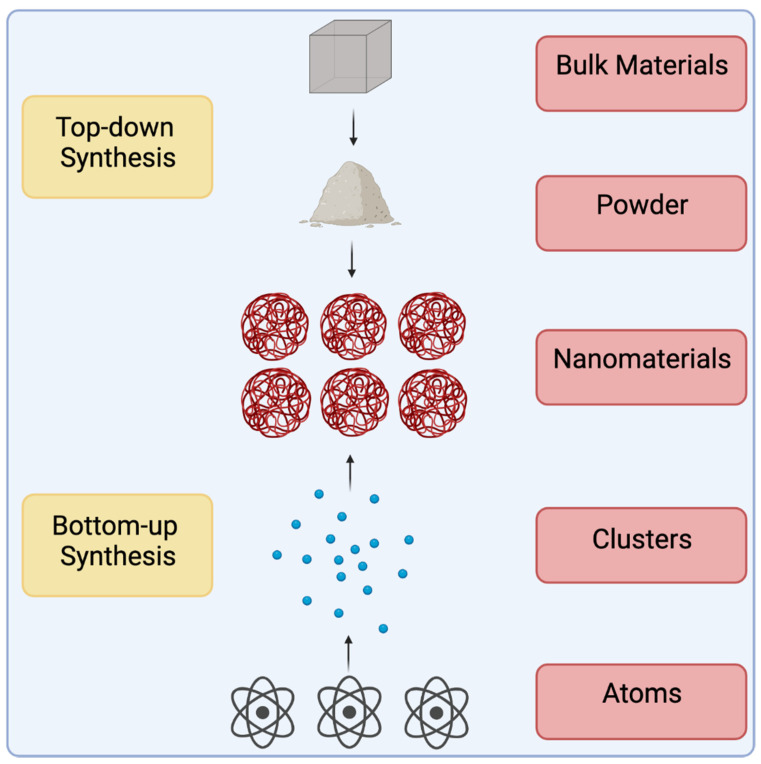
Top-down vs. Bottom-up synthesis schemes. There are two methods by which nanomaterials can be synthesized. Top-down synthesis refers to the process by which bulk materials are broken down into their monomers. Laser ablation is an example of a top-down synthesis method. Bottom-up synthesis refers to the process by which atoms are reacted with other substrates to create the desired nanomaterials. Reactions can be catalyzed by an outside force such as in hydrothermal synthesis, or the introduction of volatile compounds such as in chemical vapor deposition.

Nano-sized materials are synthesized in a multitude of ways, but there are generally two approaches to their creation: top-down and bottom-up synthesis (Figure 2). The former utilizes larger bulk material and breaks them down into nano-sized particles, and the latter utilizes individual atoms and builds them up into larger nanomaterials. Metal nanomaterial products such as silver (Ag), gold (Au) [4], selenium (Se) [5], cadmium sulfide (CdS) [6], lead sulfide (PbS) [7] and iron oxide (Fe_3_O_4_) [8], provide useful properties for diverse applications.

Nanomaterial synthesis can be subdivided into two main categories: traditional methods and green methods. Many attractive benefits exist with using traditional nanomaterial synthesis methods. These methods produce a large variety of nanoparticles with vast applications. Some methods offer extensive scalability [9], high control over nanoparticle morphology [10,11,12], with applications in innovative battery conduction, electrical applications [13,14,15,16,17], targeted disease therapy [18,19] and energy storage/conservation [20,21,22]. However, the outstanding negative effects of employing these traditional methods are undeniable. Organic solvents are exceedingly utilized in the synthesis of these nanomaterials, posing a major neurobehavioral and reproductive risk during synthesis [23,24,25]; additionally, the use of high pressure and heat conditions may also aid in dangerous working conditions [26,27,28]. Concern for volatile vapor [29] and excessive cardon dioxide production, which contributes remarkably to the greenhouse effect, is an adverse effect of highest priority from these syntheses [30,31]. Overall, these methods pose irreversible risks to both the scientists conducting the synthesis and the environment. These potential harms outweigh the benefit of traditional nanomaterial synthesis methods. Due to these factors, traditional synthesis methods have fallen out of favor, which has paved the way for green synthesis. With the current climate crisis, the development of new and forward-looking methods that follow the 12 Principles of Green Chemistry is of vital importance.

**Figure 3:**
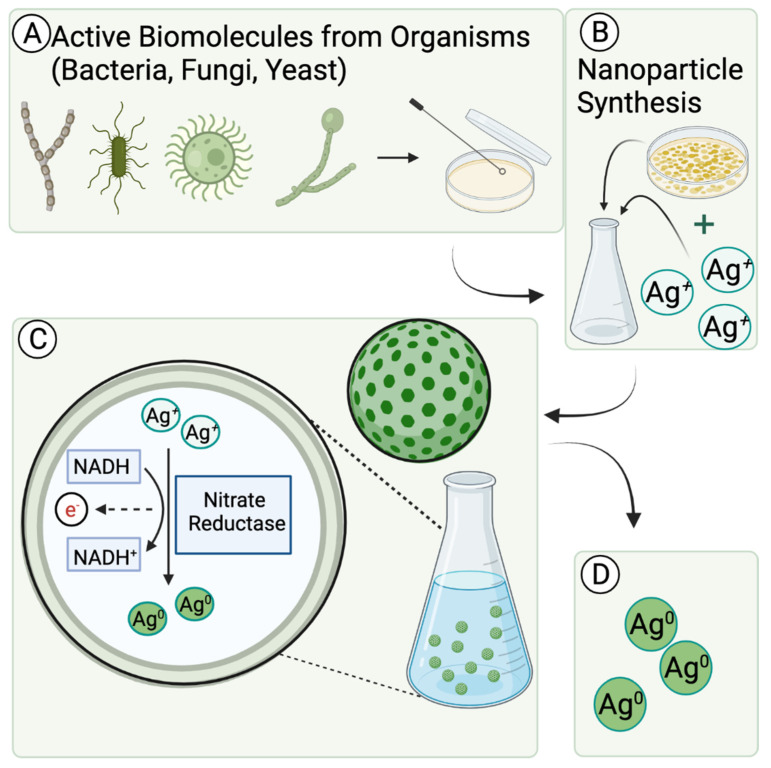
Representation of the role of active molecules in green metallic nanoparticle synthesis. (**A**) Microorganism obtained from raw sample is cultured on a plate. (**B**) Culture is harvested and purified before it is inoculated in sterile nutrient broth with metal ion solution. Methods are taken to promote homeostasis for subsequent nanoparticle synthesis. (**C**) Metal ions are reduced to metal nanoparticles, facilitated by microorganism’s active molecules. The proposed active molecule mechanism reflected in this figure is the intracellular conversion of metal ion to metal nanoparticle through an enzymatic reduction oxidation process. (**D**) Nanoparticles are collected and analyzed for purity and formation. Stability and reducing properties from microorganism can be observed in final product.

Green synthesis employs a clean, safe, cost effective and environmentally friendly process of constructing nanomaterials. Microorganisms such as bacteria, yeast, fungi, algal species and certain plants act as substrates for the green synthesis of nanomaterials (Figure 3). Different active molecules and precursors, such as metal salt, determine the final morphology and size of the nanoparticle. Additionally, green synthesis provides nanomaterial benefits ranging from antimicrobial properties [32] to natural reducing properties and stabilizing properties. The active molecules of the microorganisms utilized as green synthesis substrates attribute to these properties—a recent discovery since the last published comprehensive nanomaterial review by Saratale, R., et al. [33]. The part of the green species utilized in the synthesis of nanomaterials often consist of specific enzymes [34], amino acid groups [35], proteins [36], or chemical structure [37].

In this review, we highlight traditional synthesis methods and applications in addition to green synthesis methods and applications of nanomaterials. We pay specific attention to the active molecules produced by a variety of microorganisms that attribute to specialized nano-sized material production. Substantial benefit exists in pinpointing these active molecules as they are responsible for determining the specific morphology, size, and application of nanoparticles produced. Understanding the role of active molecules in nanomaterial production can pave the way for manipulation of these natural chemical properties to continue nanostructure advancements in the scientific community. Overall, the role of active molecules provides a more refined insight into the capability of nanoparticle synthesis from green synthesis methods in future applications.

## 2. Traditional Synthesis Methods

### 2.1. Sol-Gel Synthesis

Sol-Gel synthesis is a common method for the synthesis of nanomaterials. This relatively simple method is straightforward and can be easily utilized for the synthesis of nanomaterials using a variety of different metal oxides such as TiO_2_, ZnO, SnO_2_, WO_3_, Fe_2_O_3_ as well as silica and Platinum [38,39,40,41]. This process usually progresses over a series of five steps, beginning with hydrolysis of the precursors using either water or an organic solvent. Next, molecules that are adjacent to one another begin to form linkages as the process continues into the condensation step. The resulting “gel” is then aged and dried by supercritical drying, thermal drying, or freeze drying, with each producing slightly different products. Finally, calcination is performed in order to drive off residues and dry any remaining water [38].

Nanomaterials synthesized using Sol-Gel have wide-spread applications including drug delivery, wastewater treatment, construction materials, and a variety of sensors [41,42,43,44,45,46,47,48]. This method can be applied at an industrial level due in part to the limited number of ingredients that are required to facilitate the final product [49,50]. Additionally, Sol-Gel synthesis can progress utilizing only one-pot which adds to its allure [44].

As effective as the Sol-Gel synthesis method can be for the manufacturing of nanomaterials, it has several shortcomings regarding environmental and personal safety. Firstly, organic solvents that are typically used for the hydrolysis of the nanomaterial precursors pose enormous health and environmental risks [23,24]. It has been shown that organic solvents can affect a variety of different bodily systems, including neurobehavioral and reproductive systems [23,25,51]. Although this method is effective and efficient, it poses significant risks that cannot be overlooked.

### 2.2. Chemical Vapor Deposition

In general, Chemical Vapor Deposition (CVD) is the process by which a substrate, such as Nickel, Iron, or Zinc is introduced to one or more volatile compounds (vapor or gas) which react with the substrate, to produce a final 2D product [8,52,53,54,55]. The reaction between the substrate and volatile compound is contained within a vacuum, conducted at high temperature, in the presence of N_2_ gas and often a catalyst [56]. The temperature, substrates and precursors can be altered in order to produce products with different morphologies, sizes, and geometries [8]. Carbon nanomaterials are just one example of the amount of control that the scientist has over the final product synthesized by CVD. Graphene, fullerene, carbon nanotubes, and diamond-like carbon films are some of the nanostructures that can be created via CVD synthesis [53,57,58,59].

Because of the wide variety of nanostructures that can be created via CVD synthesis, there also exists a wide variety of applications of the nanomaterials. Though a majority of these applications overlap with nanomaterials created by other synthesis methods, there exist some interesting and unique applications as well. In the past ten years, a number of groups have utilized slightly different versions of CVD in order to produce graphene glass [60,61,62,63,64,65]. This graphene glass can be used in transparent electrodes, windows, and touch panels, amongst other applications [60,63,64]. Other applications of CVD synthesized nanomaterials include semiconductors, nanosensors, conductive electrodes, and optics [66,67,68].

This technique of nanomaterial synthesis is quickly evolving and has several derivatives from the original method, each of which posing their own risks. One such risk that spans these slightly different techniques is the use of volatile vapors and gasses. Though the gas or vapor in and of itself is not necessarily harmful, the side products that are produced as a result of the reaction with the substrate or catalyst often are harmful to both the environment and individual conducting the synthesis [29]. Plata et al. identified over 45 different compounds that are produced as a result of synthesis of carbon nanotubes [69]. Although these side products are worrisome, they are not the primary risk associated with this method. CVD synthesis requires large amounts of energy to heat the vacuums to their final temperature (~1000 °C) [70].

### 2.3. Hydrothermal Synthesis

Hydrothermal synthesis, also known as solvothermal synthesis, is an overarching term for techniques that take advantage of materials solubility by placing them under intensely hot water and high pressure, which results in crystalline structures. To form the products, precursors, water (or other solvent) and stabilizing agents are combined in a steel autoclave, which is heated and left to run for a predetermined amount of time. To change the morphology, size and geometry, the individual operating the autoclave can change the precursors, alter the temperature and/or change the pH of the solution in the autoclave. Upon completion of the autoclave cycle, the products are then cooled to room temperature, washed, and then finally dried [71,72,73].

As with other methods of nanomaterial synthesis, structures that are produced as a result of hydrothermal synthesis have applications in a wide variety of sectors and the materials applications are largely based upon their size, morphology, geometry and surface coatings. One of the more exciting applications of hydrothermal synthesis is the manufacturing of the components of Na-ion and K-ion batteries. Specifically, the hydrothermal method is utilized in the synthesis of different nanostructures such as nanorods (non-conductive), nanowires (conductive), and nanosheets for the electrodes of the batteries [13,14,15,16,17]. Beyond electrical applications, nanomaterials synthesized via hydrothermal synthesis can be applied in a number of different ways in healthcare, sensing devices, and electric media storage, amongst others. Darr et al. produced a comprehensive review of the applications of hydrothermal produced nanomaterials that covers this more extensively [74].

Compared to other methods of nanomaterial synthesis, hydrothermal synthesis is much cleaner and more energy efficient, which is achieved primarily through the use of lower temperatures in the autoclave [30,75,76]. Although this method is a step in the right direction for large-scale production of nanomaterials, it does not fully satisfy the 12 Principles of Green Chemistry. Caramazana et al. reported that the process of hydrothermal synthesis produced 10.86 kg of CO_2_ per kg of Ag_2_S nanoparticles [30] which is significantly less compared to 543 kg of CO_2_ per kg of Ag_2_S nanoparticles produced by flame spray pyrolysis [31].

### 2.4. Ultrasound Synthesis

Ultrasound, or sonochemistry, is a common laboratory technique used for the creation of nanomaterials. This technique manipulates acoustic waves to cause cavitation events, which in turn drive chemical reactions that result in the growth of nano-scale materials. Cavitation is the process by which microbubbles in a liquid rapidly store ultrasonic energy, grow, and subsequently collapse, releasing the ultrasonic energy back into the environment. Cavitation event is restricted and generate intense heat (5000 K) and pressure (1000 Bar) for a very short amount of time [77,78,79,80,81]. The cavitation event produces an interaction and/or a chemical reaction with a precursor in the environment, while the chemical reaction produces the final nanomaterial [78]. The size and morphology of the produced nanomaterial can be controlled by varying the precursors, the liquid in which the reaction takes place, and the frequency of the ultrasound waves.

The precursor of the nanocomposite is crucial to what it can be applied to; transition metal carbides, for instance, are highly effective precursors for the creation of nanoparticles with applications in chemical refinement and uses, including magnets. Mo_2_C and W_2_C nanoparticles are highly effective catalysts for hydrodehalogenation and are used to keep our bodies and environment clean from dangerous chloroflourocarbons and other hazardous halogenated organic chemicals [82,83,84]. Sonochemically synthesized nanomaterials can also be utilized as magnets in a variety of applications, including drug delivery. The drug attached to a magnetic nanoparticle is transported through the body using local magnets, placed at the site that the drug is required [85,86,87].

Ultrasound/sonochemistry requires little to no organic solvents or other harsh chemicals and is one of the most environmentally friendly methods of “traditional” nanomaterial synthesis. Additionally, the energy required for production of ultrasonic waves is meager compared to the energy required for other systems such as flame spray pyrolysis and Sol-Gel synthesis [77,88]. However, one shortcoming of ultrasonic method is its scalability, and large-scale ultrasonic synthesis that does not require chemical catalysts is under investigation. Recently, Hujjatul Islam et al. determined that large-scale synthesis of nanoparticles was possible by using an ultrasonic one-pot synthesis method [89]. This is just one of many steps that open the door for large-scale synthesis of nanomaterials.

### 2.5. Laser Ablation

Laser ablation is the process by which bulk material is broken down by laser pulses into its smaller constituents. The constituents that are released during the ablation are nano-sized and are collected as the final product. This process takes place in either a gaseous or liquid medium to control the size and shape of the resulting nanomaterial. In gas or a vacuum, the nanomaterial collects on a surface as a thin film. In contrast, when performed in a liquid, a colloidal structure is formed [90]. Further, by altering the intensity, pulse length and wavelength of the laser, the user is able to control the size and shape of the final product. The laser can also be utilized to further “fine tune” the shape and size of the nanomaterial, giving the user immense control over the final product [10,11,12].

Recently, it was identified that laser ablated nanomaterials are functional in the treatment of a number of diseases, namely, certain cancers. Walter et al. determined that AuNP generated by laser ablation in Tris buffer, with conjugated aptamers, were effective in the detection of human prostate cancers [18]. Additionally, Salmaso et al. were successful in detecting human breast adenocarcinoma in culture with laser ablated AuNP with a thermoresponsive polymer coating [19]. Beyond cancer, laser ablation synthesized nanomaterials can be applied to luminescent semiconductors, biosensing and imaging, and as nanofertilizers for seed germination [11,91,92,93].

Though laser ablation can be utilized in a “greener” fashion, it is often coupled with organic solvents in order to further control the morphology of the product, which is often the case for a variety of metal nanoparticles [94,95,96]. As previously mentioned, organic solvents are not only dangerous for the individual using them, but are harmful to the environment as well [23,25]. Further, laser ablation is one of the most energy consuming methods of nanomaterial synthesis, which has secondary effects on the environment, as coal and gas are often used to generate the electricity consumed in powering the laser [97].

### 2.6. Flame Spray Pyrolysis

**Figure 4:**
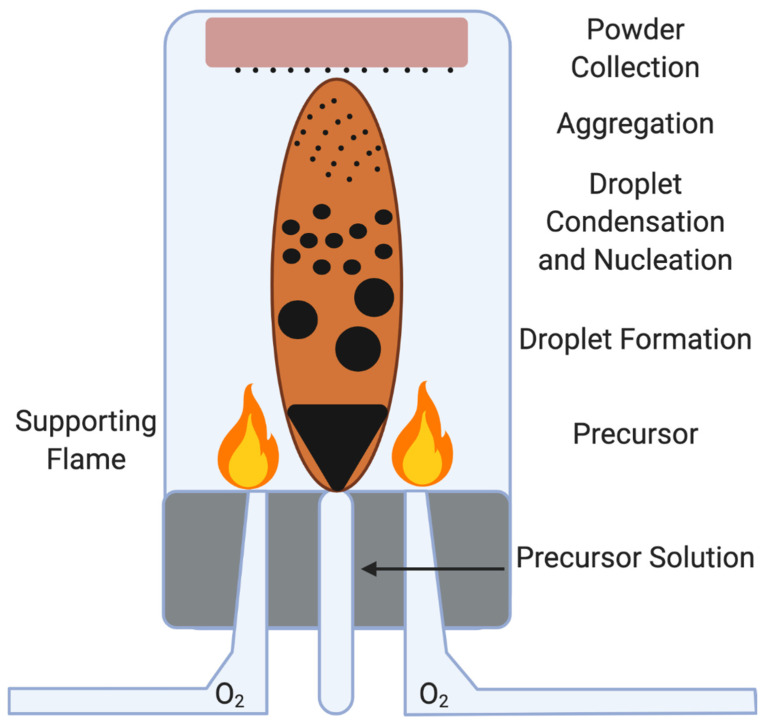
Nanoparticle formation by Flame Spray Pyrolysis. Flame spray pyrolysis is a complicated process that involves the breakdown of a precursor (typically an organic solvent) into its monomers, which are then reacted with hydrocarbons and catalyzed by very high temperatures. The resulting nanomaterials are collected on substrate. FSP is scalable to industrial levels, but is highly dangerous and the carbon dioxide biproducts are major contributors to the greenhouse effect.

Flame spray pyrolysis (FSP) is a process by which nanomaterials are synthesized by combining a high enthalpy precursor (typically an organic solvent) with oxygen and hydrocarbons in a flame [73]. Once these ingredients mix and nanomaterials begin to form, they pass through a filter and are collected on a substrate (Figure 4). The size, shape and morphology of the final product can be controlled by modulating the precursor, oxygen level and temperature via amount of hydrocarbons released into the environment. One of the more important facets to creating the desired nanomaterial is the precursor selected. To produce a homogenous morphology of nanomaterials, precursors with high enthalpies and low melting points must be selected; otherwise (without fine-tuning the processing conditions), the final products will be a heterogenous mixture of nanomaterials that are largely unusable. This process can be dangerous to the individual(s) operating the furnace as temperatures inside the furnace can reach as high as 2800 K [26,27,28]. Further, this process is scalable and is done on an industrial level [9].

Versions of FSP have been around for decades and as such, a diverse field of applications have been discovered [28]. One such application are catalysts, such as photocatalysts (from TiO_2_ and ZnO), CO oxidation catalysts (from Au/TiO_2_) and dehydrogenation catalysts (from Pt-Sn/Al_2_O_3_), amongst others [27,98,99]. Further, Eckert et al. determined a low-cost manner to produce metal oxides through FSP that have applications in lasers [100]. Beyond catalysts and lasers, nanomaterials synthesized by FSP have applications in energy storage and energy conversion, solar cells, and dye degradation [20,21,22].

FSP is, without a doubt, one of the most environmentally harmful and dangerous methods of nanomaterial synthesis. According to Eckelman et al., FSP produces over 50× more carbon dioxide as compared to a hydrothermal approach [31]. Different methods of FSP utilized for nanomaterial synthesis can have different adverse environmental and personal effects. For instance, the basis of FSP is the burning of hydrocarbon fuel in order to create the environment for nanomaterial creation. The burning of hydrocarbons produces carbon dioxide, which is the primary factor in the greenhouse effect [101,102,103,104]. Further, FSP commonly uses organic solvents to contain the precursors which are harmful to the body and environment.

## 3. Green Synthesis

### 3.1. Background

As a population of scientists, we can no longer allow green science to be another avenue which we can take, but rather, must be the avenue. As the scientific communities’ knowledge of the synthesis of nanomaterials grows, so do the means by which they can be created without harming the Earth and the people who live on it.

The basis of green chemistry was established in the early 2000s by Anastas and Warner with their book, “Green Chemistry” in which they published the 12 Principles of Green Chemistry [105]. The Principles are as follows:

1. Prevention—Steps must be taken to prevent the production of waste.
2. Atom Economy—As much as possible, the materials used in the synthesis should be incorporated into the final product.
3. Less Hazardous Chemical Synthesis—Synthesis methods that require materials with minimal or no toxicity to the environment or individual should be prioritized.
4. Designing Safer Chemicals—Chemicals should designed to achieve function with limited or no toxicity.
5. Safer Solvents—The use of solvents and auxiliary chemicals should not be used when possible.
6. Design for Energy Efficiency—Energy usage should be limited for synthesis.
7. Use of Renewable Feedstocks—a feedstock should be renewable and depletion should be avoided whenever possible.
8. Reduce Derivatives—Derivatives such as blocking agents and protecting/deprotecting groups should be avoided whenever possible as they cause additional waste.
9. Catalysis—Catalysis agents are preferable to stoichiometric agents.
10. Design for Degradation—Chemicals should be designed so that at the end of synthesis, they will break down into non-toxic derivatives.
11. Real-time Analysis for Pollution Prevention—Synthesis should be monitored in real-time for toxic chemical production.
12. Inherently Safer Chemistry for Accident Prevention—Agents used in product synthesis should be selected to limit the possibility of hazardous accidents.

These 12 principles should be followed whenever possible in order to limit the release of hazardous materials into the environment as well as human exposure to these chemicals [105]. These 12 principles were expanded upon in 2012 by Gałuszka et al. in a review of green analytical chemistry [106]. In this review, the group proposed the mnemonic “SIGNIFICANCE” as an easy way to remember the 12 Principles of Green Chemistry.

- S—Select direct analytical technique
- I—Integrate analytical processes and operations
- G—Generate as little waste as possible and treat it properly
- N—Never waste energy
- I—Implement automation and miniaturization of methods
- F—Favor agents obtained from renewable source
- I—Increase safety of operator
- C—Carry out in-situ measurements
- A—Avoid derivatization
- N—Note the sample number and size should be minimal
- C—Choose multi-analyte or multi-parameter methods
- E—Eliminate or replace toxic reagents

A key point that both of these groups touch on is the use of agricultural waste as reducing and capping agents. This waste often takes the form of different plant materials such as onion peels, banana peels, honey, and many other waste products [1,107,108,109,110]. Further, many nanomaterials are synthesized using different solvents, many of which (as previously noted) are of an organic nature; however, this does not have to be the case, as it is possible to utilize H_2_O to form nanomaterials. Water, even when slightly heated, can form nanomaterials. Additionally, when the water is heated to a supercritical state (the state in which it is above its critical temperature and pressure, but below that to become a solid, its properties change and it is able to function as a solvent in the reaction [111,112,113]). Other compounds that can be used as supercritical fluids for nanomaterial synthesis include ethanol and carbon dioxide [112,114].

There are many different facets of nanomaterials that play into their efficacy in different systems. For instance, silver nanoparticle coated nanosheets are often utilized in electronics as they have highly sought after electrical catalytic potential ([115]). However, these same sheets would not be effective in biomedical imaging techniques, such as MRI. Thus, the morphology, size, and material which the nanomaterial is composed of are incredibly important in the function of the nanomaterial.

There are typically three general types of morphologies that are recognized as nanomaterials, each of which build on the previous, with 0D being the first, 1D the second, and 2D the third. The “dimensions” refer to any part of the nanomaterial that stretches beyond the 100 nanometer range and sometimes into the micrometer(s). Typically, 0D nanomaterials are nanoparticles that can take a variety of shapes, from triangles and hexagons to spherical, polymeric forms. The 0D nanoparticles are often utilized in drug delivery, medical imaging, and other biomedical applications. However, they also have optical and electronic applications, amongst others. In terms of 1D nanomaterials, these typically take the form of nanorods, nanowires, or nanotubes. These materials vary slightly in their formation and the materials they are made of. For instance, nanotubes are typically comprised of carbon, and are formed from graphene sheets that wrap into a cylinder. The last type of nanomaterials are 2D. These nanomaterials have two dimensions that stretch beyond the 100 nanometer mark and include graphene, nanofilms, and nanocoatings. Each of these nanomaterials can be synthesized using green substrates and techniques.

### 3.2. 0D Nanomaterials

A large portion of nanosystems, regardless of their final form, begin as 0D nanomaterials. These systems can be synthesized using a number of different methods and substrates such as bacteria, yeast, fungi, plant material, live plants, viruses, and pure enzymes, amongst others (Table 1, Table 2, Table 3, Table 4 and Table 5). Each of these substrates require slightly different methods in order to properly synthesize the nanomaterial.

#### 3.2.1. Bacteria

Bacteria that can be utilized in the green synthesis of nanomaterials belong to a large group of unicellular organisms with cell walls, but lack organelles and an organized nucleus. Although some strains of bacteria can be very dangerous, many strains occur naturally in the body and pose little to no harm to someone working with them. Further, many of these strains, such as *Escherichia Coli* (*E. coli*) and *bacillus subtilis*, are very easy to culture and their genetic code can be easily altered. Because of these characteristics, nanoparticle synthesis in bacteria is a feasible process. To utilize bacteria for the synthesis of nanomaterials, bacteria is first grown aerobically to a desired optical density and then the growth medium containing the cells is combined with the nanoparticle precursor. After an incubation period, and a visible media color change, the media is centrifuged at high speeds (≥10,000 RPM). The supernatant from this spin contains a suspension of the nanomaterials [4]. Different strains of bacteria and precursors determine the final morphology and size of the nanoparticle (Table 1). For instance, Gurunathan et al. and Sweeney et al. reported synthesis of different shapes of silver, gold, and cadmium nanoparticles due to their interaction with different biomolecules. Gurunathan et al. reported that the proteins on the exterior of the cell wall of *E. coli* interacted with the silver nitrate and choloauric solution to produce irregular and triangle morphologies of silver and gold nanoparticles [4]. 

However, in the synthesis of cadmium nanoparticles, glutathione and cysteine desulfhydrase in the interior of the *E. coli* were primarily involved in the formation of spherical morphology [4]. Intracellular vs. extracellular and the interaction with bioactive molecules can also be linked to the final size of the nanoparticle. In extracellular synthesis using bacteria, nanoparticles are typically larger than those synthesized intracellularly. Nanomaterials made of selenium, ferrous oxide, zinc sulfide, and lead sulfide have been synthesized using bacterial systems (Table 1). Nanoparticles can be stabilized and reduced by a number of different active molecules common in living organisms. For bacteria, amino acids of proteins present on the cell wall and in the cytosol such as tyrosine and tryptophan are able to reduce the nanoparticles and keep the stable. Further, sugars such as aldose and ketose can serve as reducing/stabilizing agents. Specifically, the amino acids in the cells walls and inside the cells function as a protective capping layer, which renders them non-toxic to mammalian cells [116]. These active molecules that exist in and on different bacteria species react with the metal ions and reduce them, allowing the metal ions to react with one another to facilitate the creation of higher ordered structures such as spherical nanoparticles.

#### 3.2.2. Yeast (Live and Extract)

Similar to bacteria, yeast are unicellular organisms and belong to the fungus family. The most traditional and common use for yeast is with the species *Saccharomyces cerevisiae*, which converts carbohydrates to alcohols and carbon dioxide. This specific species is used for baking and in alcoholic beverage creation through a process known as fermentation. Not all species of yeast are benign, such as those used in baking; some species, such as *Candida albicans*, can cause life-threatening systemic and bloodstream infections [132].

The use of yeast cells allows for the synthesis of some different nanosystems than bacteria. Silver, gold, cadmium sulfide, lead sulfide, ferrous oxide, selenium and antimony nanoparticles have all been synthesized using yeast species (Table 2). With more common nanomaterial composites, such as silver and gold, nanosystems can be synthesized using live cells, or cell extracts. Sivaraj et al. reported successful synthesis of silver chloride nanoparticles with proteins extracted from commercial yeast. To form the nanoparticles, the group began by treating commercial yeast extracts with precursor solutions and allowing to incubate for 24 h. After this incubation, the group collected the solution and sterile filtered it to obtain a solution containing only the nanoparticles. The group also showed that, specifically, the primary amine of certain proteins imbedded in the cell wall of the yeast were responsible for the reduction of the silver chloride into nanoparticles. This particular nanoparticle was also shown to have advantageous anti-mycobacterial properties [32]. In an early report, Kowshik et al. showed nanoparticles synthesized by silver-tolerant *MKY-3* yeast cells [133]. These nanoparticles had different morphologies and sizes, depending on their synthesis conditions (concentration of silver chloride, pH, etc.). This particular study suggests that excreted biochemical reducing agents were responsible for the extracellular reduction of silver chloride [133]. However, extracellular synthesis is not the most common method of nanoparticle synthesis by yeast. Most other groups reported that synthesis, in their models, occurred intracellularly and enzymes within the cell were responsible for the creation of the nanosystem. Kowshik et al. followed up their prior work by reporting that *Schizosaccharomyces pombe* and *Torulopsis* sp. were able to intracellulary synthesize cadmium sulfide and lead sulfide nanoparticles, respectively. Differing from their prior work, Kowshik et al. showed that species of phytochelatin synthase was responsible for the intracellular synthesis of the nanoparticles [134]. Interestingly, almost all the publications analyzed reported that the nanoparticles they synthesized functioned exceptionally well in different biomedical applications. The broad term “biological applications” results from the wide variety of different ways that nanoparticles can be utilized. For instance: *Saccharomyces cerevisiae* produces spherical silver nanoparticles that effectively eliminate mycobacteria in culture [32]. Similar to other living organisms, yeast are capable of producing proteins with specific amino acids that are able to reduce and stabilize the nanoparticle. Specific to yeast, quinones are organic molecules derived from aromatic compounds and are reported to assist in the production of nanoparticles. The oxidoreductases are activated when the internal pH becomes more basic and subsequently allows them to reduce the metal ions. The quinones are strong nucleophiles with redox properties that are suitable to facilitate the conversion from simple metal ions to higher ordered nanoparticles [5,135].

#### 3.2.3. Fungi

An umbrella term that technically includes yeast, fungi are eukaryotic organisms that acquire their food by secreting digestive enzymes into their immediate environment, then absorbing the dissolved molecules. However, their defining characteristic is the chitin, a long-chain polymer and derivative of glucose, that reinforces their cell walls. The cell walls of fungi, in addition to containing chitin, can also facilitate the formation of nanoparticles of different shapes, sizes, and compositions. Production of nanoparticles can be undertaken both intracellular and extracellular by enzymes and protein residues. To form nanomaterials from fungi, the fungi is first retrieved and incubated in broth, shaken for 72 h to produce a biomass which is then filtered. After extensive washing, the biomass is incubated with the nanoparticle precursor and the resulting solution, after 24 h, contains the nanoparticles [141]. Mukherjee et al. reported that *Verticillium*, a genus that is most commonly known for the Verticillium Wilt that can decimate crops worldwide, can form silver nanoparticles on the cell wall by reducing aqueous silver nitrate [142]. Sanghi et al., Ingle et al., and Gade et al., amongst others noted in Table 3, showed extracellular silver nanoparticle synthesis by different fungal species [143,144,145]. Additionally, it has been shown that intracellular proteins can facilitate nanoparticle synthesis. Ahmad et al. and Gericke et al. showed that intracellular enzymes of *Trichothecium* and *Verticillium luteoalbum*, respectively, form gold nanospheres and nanorods [146,147]. Similarly to nanoparticles formed by other green methods, nanoparticles formed by fungi have applications in many different fields, ranging from medicine to optoelectronics [34,148]. Medical and therapeutic applications of nanoparticles are exciting applications that warrant future study. Phillip et al. [127] reported that edible mushroom extracts have chemotherapeutic properties, amongst other helpful characteristics, stating that nanoparticles synthesized from these extracts carry similar properties. Phillip et al., amongst others, also reported that amino acid residues in fungi, such as cysteine, are able to assist in the production of nanoparticles [127]. Similarly to the bacteria, the amino acids in the cell wall of the fungi function as capping and stabilizing agents. Further, when the nanoparticles are applied to therapeutics, as referenced by Phillip et al., they are non-toxic, which is not the case for a traditionally synthesized nanoparticle.

#### 3.2.4. Algal Species

Algae are a group of phytosynthetic, eukaryotic organisms that are not typically considered to be plants. The chlorophyll containing single, or multicellular (depending on the species) organisms, grow on water but lack true stems, leaves and vascular tissues that characterize plants. Additionally, their effects on humans can range from therapeutics like *Spirulina*, which contains a high concentration of natural nutrients [167], to species that are lethal if their cells or toxins are ingested, such as *Anabaena* [168]. In recent years, a considerable amount of algal species have been identified for their abilities to catalyze the synthesis of nanomaterials (Table 4). To form nanomaterials from different algal species, the samples are first thoroughly dried then ground into a fine powder, added to water, incubated for 24 h and filtered. Once filtered, the biomass filtrate is combined with the nanomaterial precursor and incubated at room temperature until the color of the solution changes, indicating the formation of the nanomaterial. Similar to fungus and bacteria, some algal species, such as *Tetraselmis kochinensis,* facilitate the formation of gold nanoparticles intracellularly, through enzymes on the cell wall and in the cytoplasm [169]. However, comparing the two classes of agents, algal species, on average, have greater diversity in the morphology of nanoparticles that they can create. For instance, species such as the *Cystophora moniliformis* [170], *Scenedesmus* sp. [171], and *Leptolyngbya valderianum* [172] all form spherical nanoparticles, which is consistent with fungus and bacterial catalyzed nanoparticles. Beyond nanospheres, the Sinha et al. showed that *Pithophora oedogonia* is capable of synthesizing cubical and hexagonal silver nanoparticles [173]. Beyond morphology, algae and other green agents share similar bioactive molecules. A large majority of the nanoparticles synthesized by algal species that were recorded in Table 4 are reduced/stabilized by enzymes/proteins in their cytosol or on the membrane. Finally, a majority of nanoparticles that are synthesized by algal species function as potent bactericides. As for the bioactive molecules involved in the production of nanoparticles, algae utilize the same, but also slightly different molecules from other classes. In algae, polysaccharides, as well as protein residues, are able to reduce and stabilize the nanoparticles. One major bonus of employing algae is the presence of a wide variety of phytochemicals. Amino acids, alkaloids, carbohydrates, flavonoids, saponins, sterols, tannins, and phenolic compounds are all present in certain algae species such as *sargassum tenerrimum.* Each of the compounds, once purified, can function in their own way to further manipulate the size, shape, and active properties of the nanomaterial. This characterization by Kumar et al. paves the way for future work and applications of algae in nanomaterial synthesis [174].

#### 3.2.5. Plant and Plant Extract

**Figure 5:**
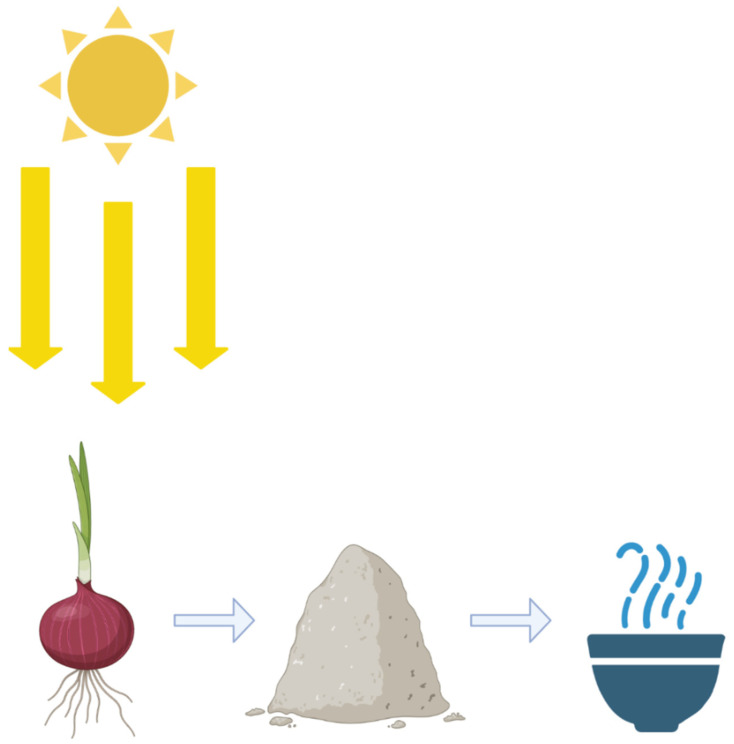
Creating nanoparticles from plant material. The creation of nanomaterials can be an incredibly complicated process, such as when FSP is employed. However, it can also be very straightforward. By drying onion peels, then grinding them up into a powder and introducing that powder to a solution with metal substrates with heat, nanoparticles can be created.

Arguably the most interesting and most environmentally friendly form of green synthesis is by utilizing plant or food scraps to create nanomaterials. Typically, the plant or food scraps undergo a process to have certain chemical compounds extracted out of them (Figure 5). In general, the plant or food scrap is dried, ground or cut up, then emerged in hot water for a period of time, then finally filtered and stored at 4 °C [1]. 

This filtered reagent can have a number of different bioactive molecules in it depending on the specific plant or plant material that they are extracted from. Commonly, flavonoids, terpenoids, and phenols are present in plant extracts [189,190], although proteins, glucosides, and polysaccharides are also implicated in the synthesis of nanoparticles [1,191,192]. These bioactive molecules contain functional parts that act as reducing and stabilizing agents for the nanoparticle precursors. Further, this method of nanoparticle synthesis eliminates the need for harsh chemicals that pose risks to both the user and the environment. Additionally, by extracting the bioactive molecules using only heated water, the need for high energy consuming techniques is also eliminated. Because of the lack of harsh reagents, a majority of the synthesized nanoparticles can be utilized in biomedical applications.

According to cited literature, silver nanoparticles are the most common type of nanoparticle synthesized by plant material (Table 5). 

Besides silver, copper, gold, and selenium have also been reported for the production of nanoparticles. The synthesis of silver nanoparticles can be accomplished by a number of different plant materials with reducing, stabilizing and capping agents present in their extracts. For instance, in addition to the agents mentioned in the first paragraph, tea polyphenols, vegetable oil, *Carpesium cernuum* (flora native to China), cannabis sativa, and black currant (a berry native to northern Europe and Asia) amongst many others, can function as reducing, stabilizing, and capping agents. Moulton et al. reported in 2010 that colloidal silver nanoparticles could be synthesized using tea leaves containing polyphenols. Their method of synthesis mirrors others that have been reported. The group received tea powder (dried and ground tea leaves) in which they boiled, filtered, and added silver nitrate to produce their silver nanoparticles which was confirmed by Transmission Electron Microscopy (TEM) [108]. The group then went on to test the toxicity of the nanoparticles on biological systems through cell viability and membrane integrity assays. The results were promising, as the nanoparticles showed no toxicity and showed potential biocompatibility [108]. Another common plant material that many people have in their kitchen is vegetable oil. Kumar et al. took a slightly different approach to the green synthesis of metallic nanoparticles by utilizing free radicals that are commonly present in common household paints made from certain vegetable oils including cashew oil [193]. The group took advantage of naturally occurring free radical exchange during the oxidative drying of oils to reduce silver benzoate (a common silver salt common in silver np synthesis) to silver nanoparticles. The group also utilized alkyd resin as the protecting agent, and fatty acids and aldehydes from the oils as stabilizing agents for the reaction. The result of their reaction were silver nanoparticle-embedded paints with antimicrobial properties [193]. Another plant agent that can be used in the synthesis of nanoparticles is aloe vera. Aloe vera is commonly used in traditional medicines to alleviate a number of different symptoms; additionally, it is also commonly used today to remedy sunburns. Fardsadegh et al. successfully employed aloe vera in the synthesis of selenium nanoparticles which carried antifungal and antibacterial properties. Although the Fardsadegh group used the hydrothermal approach, which is highlighted as a classic synthesis method, this method is more environmentally friendly than others have noted, as its most substantial downfall is high energy consumption [191]. Just as the bioactivity of molecules in plant leaves can be taken advantage of, so can the activity of molecules present in our favorite spices. The *Myristica fragrans* fruit comes from a species of evergreen trees in Indonesia. This fruit, when dried and ground, produces nutmeg and mace, common spices for cooking. When the pericarp, or non-seed part of the fruit, is dried, crushed, added to water and boiled with Cupric oxide, or silver nitrate, metallic nanoparticles form. Sasidharan et al. went on to show that flavonoids, quecetin, and phenols from the mysristic fragrans were primarily involved in the stabilization and reduction of the nanoparticles. Further, they found that the silver nanoparticles were particularly effective at breaking down bacteria cell walls. Additionally, Sasidharan et al. found that the copper nanoparticles were effective as catalysts for the construction of triazole rings [194].

Beyond plant extracts, live plants (Table 6) have also been implicated in the synthesis of nanoparticles. Utilizing live plants is one of, if not the greenest methods, by which to synthesize nanomaterials. Although this method is not very common and is seemingly not as reproducible as some other methods, it has tremendous upsides if it can be mastered. The first report of synthesis of nanoparticles through a living plant came from Gardea-Torresdey et al. who showed that silver and gold nanosystems could be synthesized using living alfalfa sprouts in 2003. The group showed that the silver from silver nitrate present in the soil that the plant was grown in was transported in its original oxidation state up the shoot of the plant. At this point, the silver particles were reduced inside of the plant and formed into nanoparticles. Once the nanoparticles began to form, they conglomerated into nanowire-like systems [195]. The other living plant species that have shown to be able to synthesize nanoparticles are black mustard plants (*Brassica Juncea*) and red fescue (*Festuca Rubra*) that was observed by Marchiol et al. After growing the plants to full size, they were exposed to 1000 ppm of silver nitrate for 24 h. During this time, the plants absorbed the silver nitrate into their roots and stems. While the silver nitrate was in the plant’s system, it was stabilized and reduced by sugars (glucose and fructose), phenols, and citric and ascorbic acids, all of which are commonly present in plants. After the reduction, the silver began to form nanoparticles that were visible by TEM [196].

### 3.3. 1D Nanomaterials

Of the nanomaterials, 0D (where all dimensions of the product are below 100 nm) are the most common form of nanomaterial, since other, higher-level structures, are often composed of smaller building blocks such as nanoparticle studded nanorod for electrical conduction (discussed more in higher ordered structures section). However, the nanorods are technically not considered 0D nanomaterials and fall into the category of 1D nanomaterials as the lengths of these materials extend beyond the nanometer scale. The structures shown in Table 7 all fall into this category. Structures that are synthesized to sizes in the micron range are often built from smaller, 0D structures. For instance, Lin et al. determined that the fabrication of silver nanowires by extracts of the *Cassia fistula* leaf evolved from individual nanoparticles [204]. This, however, is not uncommon for higher ordered structures. Other groups, such as Nadagouda et al., witnessed a similar phenomenon. Specifically, they noted that the solvent media was critical for 1D nanomaterial self assembly. When water was employed as the solvent media, Ag and Pd began to form rod-like structures, whereas isopropanol yielded wire-like structures. This difference in structure is a result of the different polarity of each of the solvents and the subsequent interactions between them and the substrate. Further, the combination of multiple forms of nanomaterials can yield interesting applications. Horta-Piñeras et al. were successful in decorating silver nanowires with spherical silver nanoparticles that showed exceptional inhibition of *E. coli* [205]. This application, though common for nanoparticles, is not as common for 1d nanomaterials. Because of the diameter, length, and conductivity of metallic nanowires and nanorods, they are often utilized in electrical and optical applications [204,206,207,208].

### 3.4. Higher Ordered Structures

After nanoparticles and other 0D structures are formed from their respective reagents, there exists the possibility, given the right conditions, that they can form higher-level structures outside of the 100 nM range. Such higher order structures include nanowires, nanotubes, nanorods (1D) and nanosheets (2D). These nanosystems possess different functional properties than their less ordered relatives, such as the ability to transmit electricity, which makes them excellent electrodes in batteries [224]. Similar to nanoparticles and other one-dimensional nanomaterials, higher ordered nanosystems can also be synthesized using environmentally friendly agents such as plant material, which is the most common agent used in systems synthesis. The higher ordered structures can be composed of metals such as silver, lead and gold, as well as other elements such as carbon. Lin et al. were able to show that broth from the *Cassia fistula* was effective at functioning as a capping and reducing agent for silver nitrate. The group was able to synthesize up to 10 micrometers of nanowires between 50 nm and 60 nm diameters. The group determined that when the *Cassia fistula* broth and silver nitrate mixture is first mixed, small nanoparticles form. However, when the solution was left to shake for multiple hours, and eventually days, the nanoparticles grew onto each other in an Ostwald ripening process. When the mixture was subjected to high temperatures (>400 °C), irregular shaped nanowires formed [204]. Another green synthesis “reagent” is viruses. In 2003, Mao et al. reported the successful synthesis of Zinc and Cadmium nanowires from ZnS and CdS, respectively. The group found that the Zn and Cd originally formed quantum dots before becoming further organized into nanowires. However, the viruses that were used in the synthesis were engineered to express pVIII fusion proteins that effectively guided the formation of the nanowires [225]. Synthesis of higher ordered nanosystems has also been seen in algae, bacteria, and fungus. In algae, Parial et al. observed that when the solution containing the algal biomass and hydrogen tetrachloraurate carried a lower pH (pH of 5), the resulting nanomaterial were nanorods as compared to the nanoparticles observed at higher pH [215]. Bacteria are equally capable of producing gold nanowires. He et al. found that *rhodopseudomonas capsulata* were able to facilitate the formation of gold nanowires from nanoparticles when the concentration of HAuCl_4_ was varied. They believe that proteins from the bacteria were the major bioactive molecules involved in the nanoparticle and nanowire formation [214]. In fungi, Das et al. found that when the solution of chloroauric acid and cell-free extract of *Rhizopus oryzae* were left to incubate for more than 24 h, gold nanowires began to form as a result of Ostwald ripening. This process, however, subsequently reduced the concentration of nanoparticles in the solution [216]. A common feature of the synthesis of higher ordered structures is that they form from lower ordered structures when certain conditions are modulated.

## 4. Conclusions

The field and application of nanomaterials is expanding, and more research continues to be conducted. Traditional synthesis methods of nanomaterials (including Sol-Gel, chemical vapor deposition, laser ablation, flame spray pyrolysis, ultrasound, and hydrothermal) are harmful to the environment due to their use of toxic reagents and high energy requirements. The usage of these methods is both ignorant and negligent, considering their distressing effects on the environment during the current rise in climate change. Green synthesis methods provide minimal to no harm to the environment, or indeed to the individuals involved in their fabrication, and are equally efficacious as the traditional synthesis methods. It is our belief that nanomaterials will revolutionize our daily lives, and we have seen first-hand the power of nanoparticles with the SARS-CoV-2 vaccines. It is also our belief that humanity is heading towards disaster in regard to our uncontrolled climate change. With these two points in mind, we believe that it is critical to conduct this groundbreaking research in an environmentally conscious way. Research into green synthesis of nanotechnology will lead us to develop more impactful medical equipment, create new supercomputer conductors, and examine space, the final frontier, with revolutionized sensors. Our article reviewed the methods of green nanomaterial production with a focus on the active molecule of various microorganism substrates facilitating the synthesis. The involvement of active molecules from natural biological systems continues to broaden with the number of organisms that can be utilized. Manipulation of the active molecules in nanomaterial synthesis results in refinement and precision of nanomaterial morphology along with antimicrobial, stabilizing, reducing and capping properties. The tailoring of nanosystem synthesis can result in small 0D nanoparticles, micron long nanowires or even nanosheets for specific application. Overall, pinpointing the active molecules in the green synthesis of nanomaterials allows continual advancements and manipulation of physical and chemical properties of nanomaterials applicable to the wider scientific community. As the world continues to adjust to climate change, continuing development in green synthesis of nanomaterials is of great importance for the preservation of environmental health, energy and ethics of scientific research.

## Figures and Tables

**Table 1:** Nanoparticles produced by Bacteria.

NPs	Species	Active Molecules	Morphology/Size (nm)	References
Ag	*Bacillus cereus*	Tyrosine and tryptophan	Spherical/4–5	[35]
	*Bacillus licheniformis*	NADPH-dependent Nitrate Reductase	Irregular/50	[66]
	*Bacillus sp.* KMS2-2	Phospholipids and proteins	Spherical/18–153	[117]
	*Corynebacterium sp.* SH09	Aldose and ketose	ND/10–15	[118]
	*Corynebacterium glutamicum*	Organic molecules of cell wall	Irregular/5–50	[119]
	*Escherichia coli*	Protein amino acids on cell wall	Irregular/50	[4]
	*Morganella* sp.	Protein amino acids on cell wall	Spherical/20 ± 5	[120]
Au	*Bacillus subtilis* 168	Aldose and ketose	Octahedral/5–25	[118]
	*Escherichia coli*	Protein amino acids on cell wall	Triangles, hexagons/20–30	[4]
	*Pseudomonas aeruginosa*	Excreted cell wall reduction enzymes	Irregular/15–30	[121]
	*Lactobacillus Kimchicus*	Sugars and NADPH-dependent reductase	Spherical/5–15	[116]
	*Ureibacillus thermosphaericus*	ND	Irregular/50–70	[122]
CdS	*Escherichia coli*	Glutathione	Spherical, elliptical/2–5	[6]
	*Gluconoacetobacter xylinus*	Bacterial-cellulose nanofibers	Spherical/30	[123]
	*Lactobacillcus*	ND	Spherical/4.9	[124]
	*Rhodopseudomonas palustris*	cysteine desulfhydrase	Cubic/8	[7]
	*Rhodobacter sphaeroides*	cysteine desulfhydrase	Hexagonal/8	[125]
Fe_3_O_4_	*Aquaspirillum magnetotacticum*	ND	Octahedral prism/40–50	[126]
	*Magnetospirillum magnetotacticum*	Amino and carboxylate acids	Cubo-octohedrons/47.1	[127]
	*Magnetospirillum* magnetotacticum (MS-1)	ND	Cuboctahedral/~50	[128]
	*Shewanella oneidensis*	Proteins, reductases, quinones and electron transferase	Rectangular, rhombic, hexagonal/40–50	[129]
Hg	*Enterobacter* sp.	ND	Spherical/2–5	[122]
MnO	*Bacillus* sp.	cardiolipins	Orthorhombic/4.62	[130]
PbS	*Rhodobacter sphaeroides*	cysteine desulfhydrase	Spherical/10.5 ± 0.15	[7]
Ti	*Lactobacillus* sp.	ND	Spherical/40–60	[124]
UO_2_	*Shewanella oneidensis*	Proteins, reductases, quinones, electron transferase	(UO2-EPS)/1–5	[129]
ZnS	*Desulfobacteraceae*	Cysteine desulfhydrase	Bio-film/2–5	[131]

**Table 2:** Nanoparticles produced by Actinomycetes and Yeast.

NPs	Species	Active Molecules	Morphology/Size (nm)	References
Au	*Rhodococcus* sp.	Enzymes in cell wall and cytoplasm	ND/5–15	[136]
	*Thermomonospora* sp.	Chloroaurate reduction and capping enzyme	Spherical/ND	[137]
	*Yarrowia lipolytica*	NADH-dependent reductases and protease	Triangles/15	[138]
Ag	*Saccharomyces cerevisiae*	Primary amine of proteins	Spherical/17	[32]
	*Yeast strain MKY3*	Excreted biochemical reducing agents	Hexagonal/2–5Irregular polygonal/9–25	[133]
Cd	*Schizosaccharomyces pombe*	Phytochelatin synthase	Wurtzite-hexagonal/1–1.5	[134]
	*Yeast*	Oxidoreductase	Spherical/3.6	[139]
Fe_3_O_4_	*Yeast*	Protein with 2 amides	Wormhole-like/<100	[138]
PbS	*Torulopsis* sp.	Phytochelatin synthase	Spherical/2–5	[134]
Sb_2_O_3_	*Saccharomyces cerevisiae*	Oxidoreductase and quinone	Spherical/2–10	[135]
Se	*Magnusiomyces ingens*	Proteins, phenols, flavonoids, amino groups	Spherical/87.82 ± 2.71	[140]
	*Saccharomyces cerevisiae*	Oxidoreductase and quinone	Spherical/75–709	[5]

**Table 3:** Nanoparticles produced by Fungi.

NPs	Species	Active Molecules	Morphology/Size (nm)	References
Ag	*Alternaria solani*	NADH-dependent reductase	Spherical/5–20	[149]
	*Aspergillus fumigatus*	Proteins and proteinic components	Spherical/5–25	[150]
	*Aspergillus niger*	Nitrate-dependent reductase and quinone	Spherical/20	[145]
	*Aspergillus terreus*	NADH-dependent reductase and nitrate reductase	Spherical/15–29	[141]
	*Cladosporium cladosporioides*	ND	Spherical/10–100	[151]
	*Coriolus versicolor*	Amine and cysteine amino residues	Spherical/25–75	[143]
	*Fusarium acuminatum*	Nitrate-dependent reductase	Spherical/5–40	[152]
	*Fusarium oxysporum*	Citrate	Variable/5–15	[153]
	*Fusarium oxysporum*	Enzymes in cell membrane	ND/5–50	[154]
	*Fusarium oxysporum*	NADH-dependent reductase	Spherical, triangular/5–15	[155]
	*Fusarium semitectum*	NADH-dependent reductase	Spherical/10–60	[156]
	*Fusarium solani* USM 3799	Carboxylate, amine, cysteine amino residues	Spherical/16.23	[144]
	*Penicillium brevicompactum*	Nitrate-dependent reductase and quinone	ND/58.35 ± 17.88	[157]
	*Penicillium citrinum*	Amide amino acids	Spherical/109	[158]
	*Penicillium fellutanum*	Nitrate reductase	Spherical/5–25	[159]
	*Penicillium funiculosum*	NADH-dependent reductase	Spherical/5–10	[160]
	*Penicllium Notatum*	Citrate	Spherical/30–40	[160]
	*Phenerochaete chrysosporium*	NADH-dependent reductase	Spherical, oval/34–90	[161]
	*Phoma glomerata*	Proteins	Spherical/60–80	[34]
	*Verticillium*	Enzymes in cell membrane	Spherical/25 ± 12	[142]
	*Verticillium* sp.	Enzymes in cell membrane	Spherical/5–50	[140]
Au	*Colletotrichum* sp.	Terpenoids, polypeptides, enzymes	Spherical/20–40	[162]
	*Fusarium oxysporum*	Oxidoreductase	Spherical, triangular/20–40	[148]
	*Trichothecium* sp.	Enzymes	Triangular, hexagonal/5–200	[146]
	*Trichothecium* sp.	Enzymes	Spheres, rods/ND	[146]
	*Verticillium luteoalbum* and isolate 6-3	Enzymes	Spheres, rods/<10	[147]
Au, Au/Ag	*Neurospora crassa*	Proteins and enzymes	Spherical/32, 20–50	[163]
Au, Ag, Au-g	*Volvariella volvacea*	Amino and carboxylate acids	Spherical, hexagonal/20–150	[127]
CdSe	*Fusarium oxysporum*	Superoxidedisumutase	Spherical/9–15	[164]
Pt	*“Fusarium oxysporum f.* sp. *lycopersici”*	Hydrogenase	Triangle, hexagons, square, rectangles/10–50	[165]
Si	*Fusarium oxysporum*	Proteins	Quasi-spherical/5–15	[166]
Ti	*Fusarium oxysporum*	Proteins	Spherical/6–13	[166]
TiO_2_	*Fusarium oxysporum*	Proteins	Spherical/6–13	[166]
Zr	*Fusarium oxysporum*	Amide amino acids	Quasi-spherical/3–11	[36]
ZrO_2_	*Fusarium oxysporum*	Amide amino acids	Spherical/3–11	[36]

**Table 4:** Nanoparticles produced by Algae.

NPs	Species	Active Molecules	Morphology/Size (nm)	References
Ag	*Caulerpa racemosa*	Peptides	Spherical, triangular/05–25	[175]
	*Chaetomorpha linum*	Carboxyl groups	Clusters/03–44	[176]
	*Cystophora moniliformis*	Metabolites and phenol compounds	Spherical/50–100	[170]
	*Gelidium amansii* (live)	Carboxyl groups	Spherical/27–54	[37]
	*Gracilaria corticata*	Phenols and peptides	Spherical/18–46	[174]
	*Laminaria japonica* (extract)	Carboxy groups of alginic acid and amide groups of proteins	Spherical to oval/31	[37]
	*Leptolyngbya valderianum*	Protein	Spherical/02–20	[172]
	*Pithophora oedogonia*	Sulfate moeity of polysaccharide	Cubical, hexagonal/25–44	[173]
	*Porphyra vietnamensis*	Sulfate moeity of polysaccharide	Spherical/13 ± 03	[177]
	*Sargassum tenerrimum*	Phenols and peptides	Spherical/20	[174]
	*Sargassum muticum* (extract)	Sulfate or hydroxyl groups	Spherical/43–79	[178]
	*Sargassum wighti*	ND	ND/08–27	[105]
	*Scenedesmus* sp.	Peptides	Spherical, crystalline/15–20	[171]
	*Spirogyra varians* (extract)	ND	Quasi–spheres/35	[179]
	*Ulva lactuca* (extract)	Polyphenols	Spherical/–	[180]
Au	*Chlorella vulgaris* (extract)	Phytochemicals with hydroxyl, carboxyl and amino functional groups	Spatial array of self assembled structures/02–10	[181]
	*Padina gymnospora* (live)	Hydroxyl groups of polysaccharides in algal cell wall	Spherical/53–67	[182]
	*Padina pavonica* (live)	-	Spherical/30–100	[183]
	*Sargassum muticum*	ND	Spherical/5.42 ± 1.18	[1]
	*Sargassum wightii* (extract)	ND	Spherical/08–12	[184]
	*Stoechospermum marginatum* (extract)	Hydroxyl groups of diterpenoids	Spherical, hexagonal, triangular/19–94	[185]
	*Tetraselmis kochinensis* (live)	Enzymes on cell wall and cytoplasm	Spherical, triangular/05–35	[169]
CuO	*Bifurcaria bifurcate* (extract)	ND	Spherical/05–45	[186]
Fe	*Chlorococcum sp. MM11* (live)	Reductases and hydrosylates of polysaccharides	Spherical/20–50	[187]
Pd	*Sargassum bovinum* (extract)	Sulfated polysaccharides	Octahedral/05–10	[188]

**Table 5:** Nanoparticles produced by plant extracts.

NPs	Species	Active Molecules	Morphology/Size (nm)	Application	References
Ag	*Acadia rigidula*	ND	Spherical/22.46 ± 10.83	Antimicrobial	[197]
	*Allium cepa* L.	Flavonoids, quercetin, glucosides	Spherical/12.5	Catalyst	[1]
	*Artemisia quttensis*	ND	ND/ND	Antibacterial, anti-cancer, antioxidant	[198]
	*Artemisia Tournefortiana Rchb*	Terpenoids	Spherical/22.89 ± 14.82	Antibacterial, anti-cancer	[198]
	*Berberis vulgaris*	Terpenoids, flavonoids, carboxylic acid	Spherical/30–70	Antimicrobial	[192]
	*Cannabis sativa*	Phenols, proteins, flavonoids	Spherical/26.52	Antibacterial, anti-yeast	[140]
	*Cinnamomum Zylinicum*	ND	Spherical/10–78.9	Antimicrobial	[199]
	*Gardenia Jasminoides Ellis*	Phenols, terpenoids, proteins	Spherical/20	Antimicrobial, dye, degradation, antioxidant	[189]
	*Mentha pulegium*	Phenols, terpenoids, proteins	Anisotropic/5–50	Antibacterial, antifungal, anti-cancer	[200]
	*P. granatum* L.	Flavonoids	Spherical/10–50	Antimicrobial, dyeing, antioxidant	[201]
Ag, Cu	*Myristica fragrans*	Flavonoids, quercetin, phenols	Spherical/10–50	Antimicrobial, catalyst	[194]
Au	*Fragaria × ananassa*	Phenols, flavonoids, terpenoids	Spherical/5–31	Medical applications	[190]
	*Ribes nigrum*	Phenols, flavonoids, terpenoids	Spherical/6–44	Medical applications	[190]
	*Ribes uva-crispa*	Phenols, flavonoids, terpenoids	Spherical/8–47	Medical applications	[190]
CuO	*Adiantum lunulatum*	ND	Spherical/6.5 ± 1.5	Agriculture	[202]
Se	Aloe Vera	Polysaccharides, phenols, flavonoids, proteins	Spherical/50	Antibacterial, anti-yeast	[191]
ZnO	*Aplha Amylase*	ND	Spherical/11	Agriculture	[203]

**Table 6:** Nanoparticles produced by live plants.

NPs	Species	Active Molecules	Morphology/Size (nm)	References
Ag	*Brassica junecea*	Glucose, fructose	ND/ND	[195]
	*Festuca rubra*	Ascorbic acid, citric acid, polyphenols	ND/ND	[195]
	*Medicago sativa*	ND	Spherical/1–100	[195]

**Table 7:** 1D Nanomaterials Produced from different plant material or other species.

NPs	Species	Active Molecules	Morphology/Diameter (nm)/Length (μm)	Application	References
Ag	*Abelmoschus esculentus* (Extract)	Catalyst	Decorated nanowire/92/100–200	Sensors	[206]
	*Camellia sinensis* (Extract)	ND	Nanowire/50/1.3	Antibacterial	[209]
	*Cassia fistula* (Extract)	Alkaloids, flavonoids, or polysaccharides	Nanowire/50–60/10	Electronic and Optical	[204]
	*Mangifera indica* (Extract)	Phenols and sugars	Decorated nanowire/~70/10	Medical (antibacterial)	[205]
	*Syzygium Aromaticum* (Extract)	Eugenol	Nanowire/39/3	Electrical	[210]
	Vitamin B2	ND	Wire, rod/20-Oct/100–200	Biological	[211]
Au	*Beta vulgaris* (Extract)	Sugars and proteins	Nanowire/15/0.2–0.4	Sensors	[212]
	*Punica granatum* (Extract)	ND	Irregular wires/30–90/ND	Optical	[213]
	*Rhodopseudomonas capsulata* (Bacteria, Extract)	Surface bound proteins	Nanowire/20–30/ND	Electronics	[214]
	*Phormidium valderianum*	Polypeptides	Nanowire/32/411	Medical	[215]
	*Rhizopus oryzae*	ND	Nanowire/10/ND	Medical	[216]
C	*Chlamydomonas reinhardtii* (Algae, Live)	ND	Nanotube/20–30/2.6 ± 0.8	Electronics	[217]
	*Cocos nucifera* (Extract)	ND	Rod, bundle/ND/ND	Medical	[218]
	*Cocos nucifera* (Extract)	ND	Tube/123/1	Pb2 Ion adsorption	[219]
	*J. Regia* (Extract)	ND	Tube/15-Aug/3.6	Medical	[220]
	*Olea Europea* (Extract)	ND	SWCNT/27-31/ND	Medical	[218]
Cu	*Eucalyptus globulus* (Extract)	Oleyl groups	Nanowire/44,245/5–60	Electronic and Optical	[221]
Pd	Vitamin B2	ND	Wire, rod/20/100–200	Biological	[211]
Pt	Dextran	ND	Nanowire-like/2.2/100	Electronic	[208]
Se	*Zooglea ramigera* (Bacteria, Live)	ND	Nanorod/30–150/ND	Medical	[222]
	*Bacillus subtilis* (Bacteria, Live)	ND	Nanowire/50/5	H_2_O_2_ Sensor	[223]
Ti	Cellulose	ND	Nanowire/ND/100–500 (nm)	Electronic and Optical	[207]

## Data Availability

Data sharing not applicable.
